# Precise Point Positioning Using Dual-Frequency GNSS Observations on Smartphone

**DOI:** 10.3390/s19092189

**Published:** 2019-05-11

**Authors:** Qiong Wu, Mengfei Sun, Changjie Zhou, Peng Zhang

**Affiliations:** College of Geo-Exploration Science and Technology, Jilin University, Changchun 130000, China; sunmf17@mails.jlu.edu.cn (M.S.); zhoucj16@mails.jlu.edu.cn (C.Z.); zhangpeng18@mails.jlu.edu.cn (P.Z.)

**Keywords:** GNSS, Android smartphone, precise point positioning

## Abstract

The update of the Android system and the emergence of the dual-frequency GNSS chips enable smartphones to acquire dual-frequency GNSS observations. In this paper, the GPS L1/L5 and Galileo E1/E5a dual-frequency PPP (precise point positioning) algorithm based on RTKLIB and GAMP was applied to analyze the positioning performance of the Xiaomi Mi 8 dual-frequency smartphone in static and kinematic modes. The results showed that in the static mode, the RMS position errors of the dual-frequency smartphone PPP solutions in the E, N, and U directions were 21.8 cm, 4.1 cm, and 11.0 cm, respectively, after convergence to 1 m within 102 min. The PPP of dual-frequency smartphone showed similar accuracy with geodetic receiver in single-frequency mode, while geodetic receiver in dual-frequency mode has higher accuracy. In the kinematic mode, the positioning track of the smartphone dual-frequency data had severe fluctuations, the positioning tracks derived from the smartphone and the geodetic receiver showed approximately difference of 3–5 m.

## 1. Introduction

Precise Point Positioning (PPP) is a method for obtaining the absolute position of a single GNSS receiver using carrier phase and pseudorange observations with high-precision IGS (International GNSS Service) products to achieve centimeter-level accuracy [1,2]. To achieve this accuracy, a geodetic GNSS (Global Navigation Satellite System) receiver and antenna relatively expensive are usually required. Smartphone with positioning function is a kind of low-cost receiver widely used in transportation, agriculture, geological and environmental monitoring due to its convenience [3,4,5].

RTKLIB is an open source software package for GNSS standard and precise positioning which supports multiple satellite systems, positioning modes and data formats [6,7]. RTKLIB is widely used in navigation; for example, it can be used for automated machine guidance (AMG) and automated machine control (AMC) of mining vehicles [8], determination of position or trajectory of aircrafts [9], and navigation of marine vessels with IMU (Inertial measurement unit) [10]. RTKLIB’s PPP module can be applied to produce daily solutions for GPS time series analysis [11], and to evaluate the seismic waveforms and the co-seismic displacements derived by an earthquake [12]. Secondary development based on RTKLIB, GAMP utilizes the PPP algorithm to process multi-GNSS undifferenced and uncombined observations [13].

Previous Android smartphones merely supported the collection of single-frequency GNSS observations. Several studies had attempted to improve the accuracy of single-frequency smartphone by using different post-processing algorithms. The pseudorange double-difference algorithm can achieve the positioning accuracy within 5 m based on carrier phase smoothing of the single-frequency smartphone observations [14]. Using relative positioning method to process the data set in a fast static mode, the decimeter-level accuracy can be achieved without solving carrier phase ambiguity [15]. The single-frequency uncombined PPP algorithm had an accuracy of 0.37 m for the horizontal component and 0.51 m for the vertical component [16]. The combination of pseudorange, carrier phase and doppler observations reached a positioning accuracy of 0.6 m in horizontal direction and 1.4 m in vertical direction, which were processed using the time series differential algorithm with the SNR-dependent weighting method [17].

Currently, the application of the dual-frequency GNSS chips embedded in smartphones allows the smartphone to record dual-frequency GNSS observations, which can eliminate the first-order ionospheric delay through ionosphere-free combination. In this paper, a Xiaomi Mi 8 dual-frequency smartphone and a Hi-target geodetic receiver were applied to conduct experiment in static and kinematic modes, aiming at investigating the PPP accuracy of such dual-frequency smartphone. The dual-frequency observations of smartphone and receiver were processed by the GPS and Galileo dual-frequency PPP algorithm modified from RTKLIB [7] and GAMP [13], and single-frequency observations were directly processed by RTKLIB PPP-static mode.

## 2. Methods

A Broadcom 47755 dual-frequency GNSS chip is embedded in Mi 8, which is capable of tracking GPS L1 C/A, GLONASS L1, BeiDou (BDS) B1, QZSS L1, Galileo (GAL) E1, GPS L5, Galileo E5a and QZSS L5 signals [18]. The GPS L1/L5 and Galileo E1/E5a dual-frequency PPP algorithm will be described in detail.

### 2.1. GPS/Galileo PPP Algorithm

Using GNSS carrier phase observation and pseudorange observation, considering the receiver and satellite instrumental delays, the observation equations can be expressed as [19,20]:

(1) $$P_{r,j}^{s,Q}=\rho_{r}^{s,Q}+c\left(dt_{r}-dt^{s,Q}+d_{r,j}^{s,Q}-d_{j}^{s,Q}\right)+T_{r}^{s,Q}+\mu_{j}^{Q}\cdot I_{r,1}^{s,Q}+\varepsilon_{r,j}^{s,Q}$$

(2) $$\phi_{r,j}^{s,Q}=\rho_{r}^{s,Q}+c\left(dt_{r}-dt^{s,Q}\right)+\delta_{r,j}^{s,Q}-\delta_{j}^{s,Q}+T_{r}^{s,Q}+\lambda_{j}^{s,Q}N_{r,j}^{s,Q}-\mu_{j}^{Q}\cdot I_{r,1}^{s,Q}+\xi_{r,j}^{s,Q}$$

where $P_{r,j}^{s,Q}$ and $\phi_{r,j}^{s,Q}$ are code and phase observations, respectively; superscripts s and Q are PRN (pseudo-random noise) and satellite system, respectively; subscripts r and j (j=1,2,5) are receiver ID and carrier frequency band, respectively; $\rho_{r}^{s,Q}$ is the geometric distance between satellite s and station *r*; c is the speed of light in vacuum; $dt_{r}$ and $dt^{s,Q}$ are receiver and satellite clock offsets, respectively; $d_{r,j}^{s,Q}$ and $d_{j}^{s,Q}$ are the receiver and satellite instrumental delays of code in seconds, respectively; $\delta_{r,j}^{s,Q}$ and $\delta_{j}^{s,Q}$ are frequency-dependent carrier phase instrumental delays in meters, respectively; $T_{r}^{s,Q}$ is the zenith tropospheric delay; $\lambda_{j}^{s,Q}$ is the signal wavelength at frequency $f_{j}$; $N_{r,j}^{s,Q}$ is the integer ambiguity; $\mu_{j}^{Q}\cdot I_{r,j}^{s,Q}$ is a frequency-dependent ionospheric delay term, $\mu_{j}^{Q}$ is the frequency-dependent factor, $I_{r,j}^{s,Q}$ is the line-of-sight(LOS) ionospheric delay on the frequency $f_{j}$; $\varepsilon_{r,j}^{s,Q}$ and $\xi_{r,j}^{s,Q}$ are measurement noise, multipath error and the sum of other unmodeled errors for pseudorange and carrier phase observation, respectively. In addition, other error terms in the GNSS observation equations, such as satellite and receiver antenna phase center correction, relativistic effect, tidal load deformation (solid tide, pole tide and ocean tide), sagnac effect, and satellite antenna phase wind-up correction, have been corrected by the models in advance. 

For the convenience of description, the DCB (Differential Code Bias) and the constants $\alpha_{i,j}$, $\beta_{i,j}$ are defined as [21]:

(3) $$DCB^{s,Q}\left(s_{i},s_{j}\right)=d_{i}^{s,Q}-d_{j}^{s,Q}$$

(4) $$DCB_{r}^{Q}\left(s_{i},s_{j}\right)=d_{r,i}^{s,Q}-d_{r,j}^{s,Q}$$

(5) $$\alpha_{i,j}=\frac{f_{i}^{2}}{f_{i}^{2}-f_{j}^{2}}$$

(6) $$\beta_{i,j}=\frac{f_{j}^{2}}{f_{i}^{2}-f_{j}^{2}}$$

where subscripts i (i=1,2,5) is carrier frequency band of observations; $s_{i}$ and $s_{j}$ are signals on frequency i and j, respectively; $DCB^{s,Q}\left(s_{i},s_{j}\right)$ and $DCB_{r}^{Q}\left(s_{i},s_{j}\right)$ are satellite and receiver DCBs between signals $s_{i}$ and $s_{j}$, respectively.

Then, satellite DCBs of ionosphere-free combination are defined as [22,23]:

(7) $$DCB^{s,G}\left(IF_{L1,L5}\right)=\alpha_{1,5}DCB^{s,G}\left(L1,IF_{L1,L2}\right)-\beta_{1,5}DCB^{s,G}\left(L5,IF_{L1,L2}\right)$$

(8) $$DCB^{s,G}\left(IF_{L1,L2}\right)=\alpha_{1,2}DCB^{s,G}\left(L1,IF_{L1,L2}\right)-\beta_{1,2}DCB^{s,G}\left(L2,IF_{L1,L2}\right)$$

(9) $$DCB^{s,E}\left(IF_{E1,E5a}\right)=\alpha_{1,5}DCB^{s,E}\left(E1,IF_{E1,E5a}\right)-\beta_{1,5}DCB^{s,E}\left(E5a,IF_{E1,E5a}\right)$$

where:

(10) $$DCB^{s,G}\left(L1,IF_{L1,L2}\right)=-\beta_{1,2}DCB^{s,G}\left(L1,L2\right)$$

(11) $$DCB^{s,G}\left(L5,IF_{L1,L2}\right)=\left\{-\beta_{1,2}DCB^{s,G}\left(L1,L2\right)+DCB^{s,G}\left(L5,L1\right)\right\}$$

(12) $$DCB^{s,E}\left(E1,IF_{E1,E5a}\right)=-\beta_{1,5}DCB^{s,E}\left(E1,E5a\right)$$

(13) $$DCB^{s,E}\left(E5a,IF_{E1,E5a}\right)=\left\{-\beta_{1,5}DCB^{s,E}\left(E1,E5a\right)+DCB^{s,E}\left(E5a,E1\right)\right\}$$

where $IF_{L1,L5}$, $IF_{L1,L2}$, $IF_{E1,E5a}$ are ionosphere-free combinations of GPS L1/L5, L1/L2 and Galileo E1/E5a, respectively. The L1, L2, L5, E1 and E5a are GPS L1, L2, L5, Galileo E1 and E5a signals, respectively. $DCB^{s,G}\left(L1,L2\right)$, $DCB^{s,G}\left(L5,L1\right)$ and $DCB^{s,E}\left(E1,E5a\right)$ are provided by DCB files from CAS (Chinese Academy of Sciences).

The IGS GPS/Galileo precise orbit and clock products are obtained at IGS analysis centers by processing the ionosphere-free combination of GPS L1/L2, Galileo E1/E5a code and phase measurements, and the corresponding satellite DCBs are lump into receiver clock offsets in the procedure. Thus, when using IGS products, satellite DCBs can be ignored for Galileo E1/E5a ionosphere-free combination mode in PPP, while the satellite DCBs present in the pseudorange observation need to be corrected for GPS L1/L5 ionosphere-free combination [22,24]. At the same time, using IGS clock products introduces satellite DCBs in phase observations, and that will propagate with the phase ambiguities. If the biases are estimated as independent parameters, it will lead to a rank deficiency. Thus, we used the external satellite DCB files [25].

The receiver DCBs ($DCB_{r}^{Q}\left(i,j\right)$) can be absorbed by the receiver clock offsets ($dt_{r}$) and will not affect the parameters estimation. The carrier phase instrumental delays ($\delta_{r,j}^{s,Q},\;\delta_{j}^{s,Q}$) are related to the ambiguity, which can be absorbed by the integer ambiguity to form the floating ambiguity ($N_{r,IF_{E1,E5a}}^{s,E}$) in ionosphere-free combination. Therefore, the ionosphere-free combination observation equation can be expressed as:

(14) $$P_{r,IF_{L1,L5}}^{s,G}=\rho_{r}^{s,G}+c\left\{dt_{r}-\left(DCB^{s,G}\left(IF_{L1,L5}\right)-DCB^{s,G}\left(IF_{L1,L2}\right)\right)\right\}+T^{s,G}+\varepsilon_{r,IF_{L1,L5}}^{s,G}$$

(15) $$P_{r,IF_{E1,E5a}}^{s,E}=\rho_{r}^{s,E}+c\left\{dt_{r}+ISB_{E-G}\right\}+T^{s,E}+\varepsilon_{r,IF_{E1,E5a}}^{s,E}$$

(16) $$\phi_{r,IF_{L1,L5}}^{s,E}=\rho_{r}^{s,G}+c\left\{dt_{r}-DCB^{s,G}\left(IF_{L1,L2}\right)\right\}+T^{s,G}+\lambda_{IF_{L1,L5}}N_{r,IF_{L1,L5}}^{s,G}+\xi_{r,IF_{L1,L5}}^{s,G}$$

(17) $$\begin{array}{l} \phi_{r,IF_{E1,E5a}}^{s,E}=\rho_{r}^{s,E}+c\left\{dt_{r}+ISB_{E-G}-DCB^{s,E}\left(IF_{E1,E5a}\right)\right\}+T^{s,E}+\lambda_{IF_{E_{1},E_{5a}}}N_{r,IF_{E1,E5a}}^{s,E}\\ \;\;\;\;\;\;+\xi_{r,IF_{E1,E5a}}^{s,E} \end{array}$$

where $ISB_{E-G}$ is the ISB (Inter-System Bias) between GPS and Galileo.

The EKF (extended Kalman filter) is used to estimate the unknown parameters in RTKLIB. For the single-frequency and dual-frequency ionosphere-free combination PPP, the number of estimated parameters is equal. The unknown state vector $X_{single}$ and $X_{dual}$ can be written as follows:

(18) $$X_{single}={\left[\begin{array}{cccccc} r_{r}^{T} & cdt_{r} & Z_{r} & G_{N,r} & G_{E,r} & {N_{r,1}}^{T} \end{array}\right]}^{T}$$

(19) $$X_{dual}={\left[\begin{array}{cccccc} r_{r}^{T} & cdt_{r} & Z_{r} & G_{N,r} & G_{E,r} & {N_{r,IF}}^{T} \end{array}\right]}^{T}$$

where $r_{r}^{T}$ is receiver antenna position in ECEF frame, $Z_{r}$ is ZTD (zenith total delay), $G_{N,r}$ and $G_{E,r}$ are the north and east component of tropospheric gradients, $N_{r,1}={\left[\begin{array}{cccc} N_{r,1}^{1} & N_{r,1}^{2} & \ldots & N_{r,1}^{m} \end{array}\right]}^{T}$ is single-frequency phase ambiguity, $N_{r,IF}={\left[\begin{array}{cccc} N_{r,IF}^{1} & N_{r,IF}^{2} & \ldots & N_{r,IF}^{m} \end{array}\right]}^{T}$ is ionosphere-free phase ambiguity, m is the number of valid satellites in an epoch.

The measurement vector $y_{single}$, $y_{dual}$ for the single-frequency and dual-frequency are expressed as:

(20) $$y_{single}={\left(\begin{array}{cc} {\phi_{1}}^{T} & {P_{1}}^{T} \end{array}\right)}^{T}$$

(21) $$y_{dual}={\left(\begin{array}{cc} {\phi_{IF}}^{T} & {P_{IF}}^{T} \end{array}\right)}^{T}$$

where:

(22) $$\phi_{1}={\left(\phi_{r,1}^{1},\phi_{r,1}^{2},\phi_{r,1}^{3},\cdots ,\phi_{r,1}^{m}\right)}^{T}$$

(23) $$P_{1}={\left(P_{r,1}^{1},P_{r,1}^{2},P_{r,1}^{3},\cdots ,P_{r,1}^{m}\right)}^{T}$$

(24) $$\phi_{IF}={\left(\phi_{r,IF}^{1},\phi_{r,IF}^{2},\phi_{r,IF}^{3},\cdots ,\phi_{r,IF}^{m}\right)}^{T}$$

(25) $$P_{IF}={\left(P_{r,IF}^{1},P_{r,IF}^{2},P_{r,IF}^{3},\cdots ,P_{r,IF}^{m}\right)}^{T}$$

By using EKF, the state vector ${\hat{x}}_{k}$ and its covariance matrix $P_{k}$ at the epoch time $t_{k}$ can be estimated by:

(26) $${\hat{x}}_{k}\left(+\right)={\hat{x}}_{k}\left(-\right)+K_{k}\left(y_{k}-h\left({\hat{x}}_{k}\left(-\right)\right)\right)$$

(27) $$P_{k}\left(+\right)=\left(I-K_{k}H\left({\hat{x}}_{k}\left(-\right)\right)\right)P_{k}\left(-\right)$$

(28) $$K_{k}=P_{k}\left(-\right)H\left({\hat{x}}_{k}\left(-\right)\right){\left(H\left({\hat{x}}_{k}\left(-\right)\right)P_{k}\left(-\right)H{\left({\hat{x}}_{k}\left(-\right)\right)}^{T}+R_{k}\right)}^{-1}$$

where h(x), H(x) and $R_{k}$ are the measurement model vector, the matrix of partial derivatives and the covariance matrix of measurements errors, respectively; (−) and (+) indicate before- and after-measurement update of EKF.

For the single-frequency and dual-frequency PPP, the matrix H(x) can be expressed as:

(29) $$H\left(x\right)=\left[\begin{array}{cccc} -DE & 1 & DM_{T} & I\\ -DE & 1 & DM_{T} & 0 \end{array}\right]$$

where:

(30) $$1=\left[\begin{array}{c} 1\\ 1\\ \vdots \\ 1 \end{array}\right]$$

(31) $$M_{T}=\left[\begin{array}{ccc} M_{W}^{1} & M_{W}^{1}\cot El_{r}^{1}\cos Az_{r}^{1} & M_{W}^{1}\cot El_{r}^{1}\sin Az_{r}^{1}\\ M_{W}^{2} & M_{W}^{2}\cot El_{r}^{2}\cos Az_{r}^{2} & M_{W}^{2}\cot El_{r}^{2}\sin Az_{r}^{2}\\ \vdots & \vdots & \vdots \\ M_{W}^{m} & M_{W}^{m}\cot El_{r}^{m}\cos Az_{r}^{m} & M_{W}^{m}\cot El_{r}^{m}\sin Az_{r}^{m} \end{array}\right]$$

(32) $$D=\left[\begin{array}{ccccc} 1 & -1 & 0 & \cdots & 0\\ 1 & 0 & -1 & \cdots & 0\\ \vdots & \vdots & \vdots & \ddots & \vdots \\ 1 & 0 & 0 & \cdots & -1 \end{array}\right]$$

(33) $$E={\left(\begin{array}{cccc} {e_{r}^{1}}^{T} & {e_{r}^{2}}^{T} & \cdots & {e_{r}^{m}}^{T} \end{array}\right)}^{T}$$

where $M_{w}$ is the mapping factor of ZWD (zenith wet delay), El and Az are elevation angle and azimuth angle of the satellite from the receiver, respectively, $e_{r}^{j}$ is LOS (line-of-sight) vector from receiver to satellite.

The EKF state transition matrix is the identity matrix in PPP, and the state vector is expressed as:

(34) $${\hat{X}}_{k+1}\left(-\right)={\hat{X}}_{k}\left(+\right)$$

### 2.2. Data Processing Strategies

The single-frequency and dual-frequency observations were processed in PPP mode using the above algorithm and RTKLIB, and the parameters to be estimated included station coordinates, receiver clock offsets, zenith tropospheric delays, ISBs and the ambiguities. Orbit and clock errors were mitigated by using WUM (Wuhan University) final products, and the DCB files were from CAS. The data processing strategies are listed in Table 1.

## 3. Experiments and Results

### 3.1. Duty Cycle

**Figure 1:**
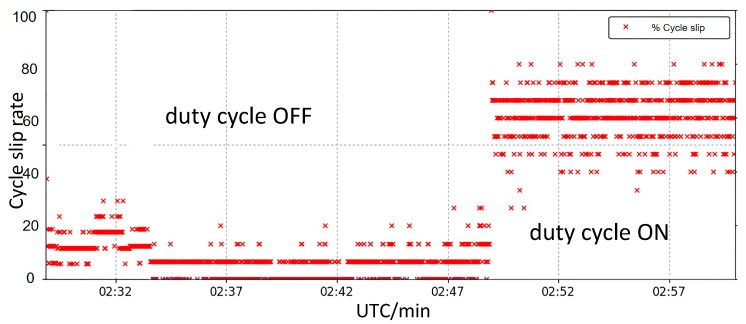
The cycle slip rate of 30-minute observations before and after turning on “duty cycle” (data collected by Mi 8 on October 7th, 2018 in the basketball court, Jilin University).

The “duty cycle” technique leads to non-continuous GNSS carrier phase tracking of smartphones [27], and a new feature of Mi 8 with the latest Android 9.0 operating system is “Force full GNSS measurements” for developers, which makes it possible to turn off the “duty cycle” when recording data [28]. After turning on the “duty cycle”, the cycle slips rate increases significantly, from about 20% to 70%, which means that turning off the “duty cycle” may increase the data availability (Figure 1).

### 3.2. Static Data Collection

**Figure 2:**
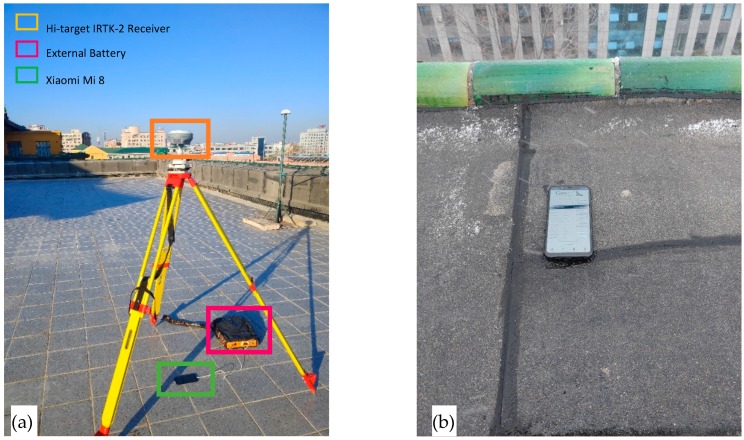
View of data collection at roof of Geological Palace Museum ((**a**) under the GNSS antenna; (**b**) on the edge of the roof).

A 24-h static data set was collected using Mi 8 and Hi-target iRTK-2 geodetic receiver with a data rate of 1 Hz on the roof top of the Geological Palace Museum of the Jilin University (43.8802° N, 125.3021° E), November 12, 2018 (DOY 316 09:20:00-DOY 317 09:20:00UTC). Figure 2a depicts the experimental equipment and environment, and we measured the distance from the bottom of the receiver to the smartphone in vertical plane and corrected it to the antenna phase center of the receiver using antenna calibrated data from manufacturer. The height between the receiver antenna phase center and phone position was 1.6939 m, the distance from the bottom of the receiver to the smartphone was 1.5997 m and the calibrated data from the bottom of the receiver to the of the antenna phase center was 0.0942 m. To avoid disturbing the other sensors, WIFI and Bluetooth were turned off, and the accelerometer, gyroscope, magnetometer and pressure were disabled during the data collection. Geo++ RINEX Logger was used to receive RINEX 3.0 data because the smartphone cannot directly record data in RINEX format [29].

**Figure 3:**
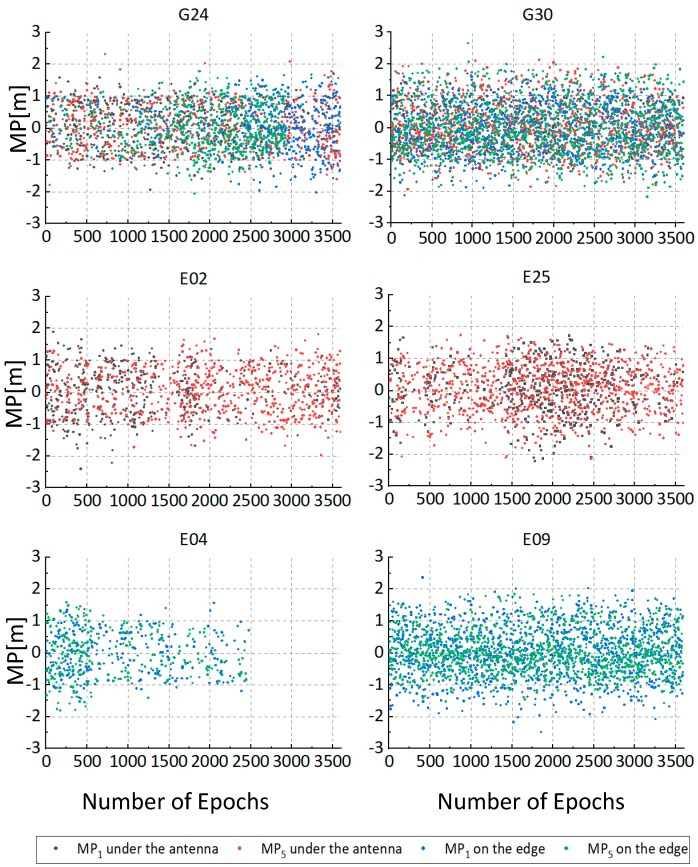
Horizontal position errors of four data sets in PPP kinematic mode.

To illustrate the validity of the arrangement of the receiver and smartphone shown in Figure 2a, an experiment was performed to evaluate the multipath effects of smartphone in different cases. We put the smartphone on the edge of the roof (Figure 2b), and compared the collected data with those placed under the GNSS antenna at the same time in the adjacent days. $MP_{1}$ and $MP_{5}$ are employed to evaluate the multipath effects, and can be expressed as:

(35) $$MP_{1}=P_{1}-\left(1+\frac{2}{\alpha -1}\right)\varphi_{1}+\left(\frac{2}{\alpha -1}\right)\varphi_{5}$$

(36) $$MP_{5}=P_{5}-\left(1+\frac{2}{\alpha -1}\right)\varphi_{1}+\left(\frac{2}{\alpha -1}\right)\varphi_{5}$$

where $MP_{1}$, $MP_{5}$ are linear combinations of pseudorange and phase observations, $P_{1}$ and $P_{5}$ are code measurements on L1 and L5, respectively, $\varphi_{1}$ and $\varphi_{5}$ are phase measurements on L1 and L5, $\alpha =f_{1}^{2}/f_{5}^{2}$ is a constant. Figure 3 and Table 2 show the multipath of the two cases, and there were no obvious differences between the two cases. The possibilities of the existence of additional multipath and the signal received by the smartphone being corrupted by the antenna were quite low.

**Figure 4:**
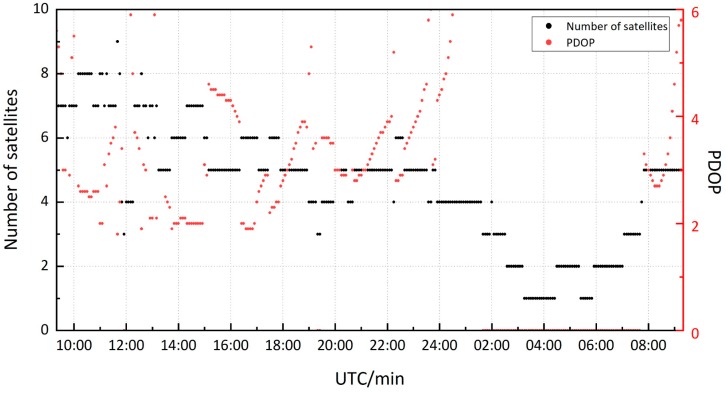
The number of satellites with simultaneous L1 and L5 frequency data record and PDOP.

The PDOP (Position Dilution of Precision) and the number of satellites recording L1 and L5 frequency data simultaneously viewed by smartphone are depicted in Figure 4. In the 24-h data collected by smartphone, the periods of more than 4 satellites were 13 h. 12 of the 31 GPS satellites and 18 Galileo satellites can transmit signals in the L5 band, and the geometric distribution of Galileo satellites are poor in the Asia-Pacific region, resulting in fewer than four satellites in many epochs. Considering the availability and continuity of data, the first six hours data (09:20-15:20 UTC) were selected for static PPP processing. In six-hour period, the number of satellites exceeded 4 in most of the time, but there were about 20 min at around 12 o’clock with fewer than 4 satellites. For the PDOP, the value was more than 4 at around 10 o’clock and 15 o’clock, and the duration of the two periods was about 40 min. The number of satellites was maintained at more than 4, and the PDOP was smaller than 4 for 4.5 h approximately. 

Four data sets were processed according to the strategies in Table 1, which included the data sets of smartphone GPS L1/L5, Galileo E1/E5a dual-frequency observations (hereafter “smartphone dual-frequency”), GPS L1, GLONASS L1, Galileo E1 single-frequency observations (hereafter “smartphone single-frequency”), and the data sets of geodetic receiver GPS L1/L5, Galileo E1/E5a dual-frequency observations (hereafter “receiver dual-frequency”), GPS L1, GLONASS L1, Galileo E1 single-frequency observations (hereafter “receiver single-frequency”). The reference coordinates were calculated by Bernese software using 12-h static data of the geodetic receiver and the data from IGS stations (e.g., BADG, BJFS, DAEJ, YSSK).

After processing, we can get the positioning result coordinates $r_{ECEF}$ and the reference coordinates $r_{r}$ in ECEF (Earth-Center Earth-Fixed). The position errors are often expressed in ENU coordinates, which could be derived from the following formula:

(37) $$r_{ENU}=E_{r}\left(r_{ECEF}-r_{r}\right)$$

where $E_{r}$ is the rotation matrix of the ECEF coordinates to the ENU coordinates and can be expressed as:

(38) $$E_{r}=\left[\begin{array}{ccc} -\sin\lambda & \cos\lambda & 0\\ -\sin\phi \cos\lambda & -\sin\phi \sin\lambda & \cos\phi \\ \cos\phi \cos\lambda & \cos\phi \sin\lambda & \sin\phi \end{array}\right]$$

where ϕ and λ are the geodetic latitude and longitude of reference coordinates, respectively.

**Figure 5:**
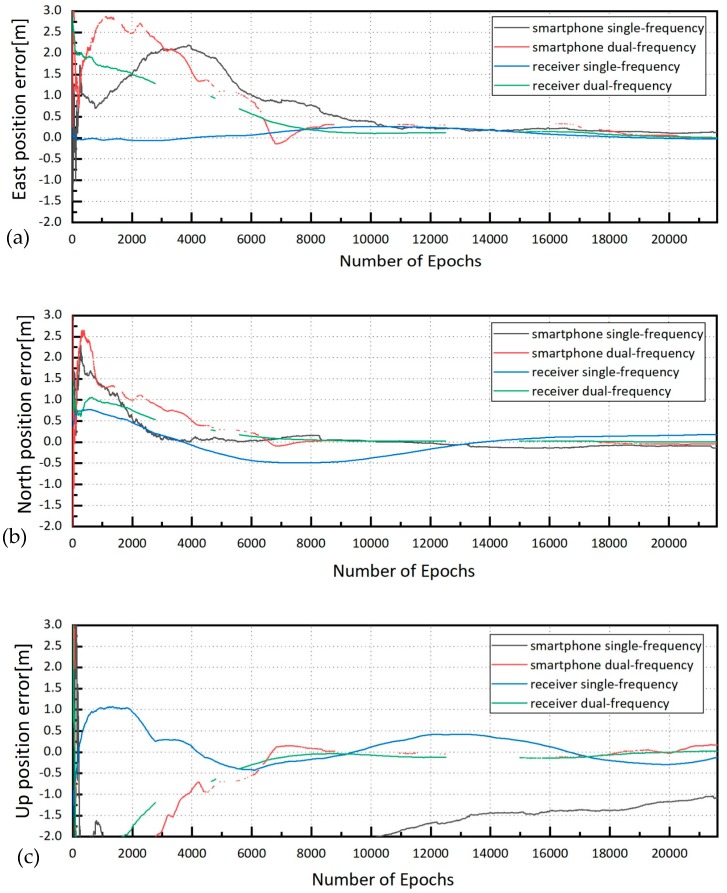
Position errors of smartphone and receiver ((**a**): east position errors; (**b**): north position errors; (**c**): up position errors).

Figure 5 showed the positioning errors of PPP solutions of smartphone and receiver. In the E direction, the positioning errors of the four data sets converged to 0.5 m at 9,000 epochs approximately, and converged to 0.2 m at 20,000 epochs (Figure 5a). In the N direction, “receiver single-frequency” showed the longest convergence time with the worst positioning accuracy (Figure 5b). Horizontal components of PPP mode from the different data sets achieved an accuracy of decimeter-level. In the U direction, obvious inconsistence existed in the four data sets (Figure 5c). The position errors of dual-frequency data sets (“receiver dual-frequency” and “smartphone dual-frequency”) showed similar trends and obtained the best results, the accuracy of “receiver single-frequency” was close to dual-frequency, and the “smartphone single-frequency” achieved the worst results.

The convergence time is defined as the time when a 3-dimensional positioning accuracy of 1 dm is reached and maintained for 20 epochs at least [30]. For smartphones, the accuracy is difficult to obtain. In this paper, we chose 1 m as the threshold of convergence because the accuracy of 1 m can meet the needs of most non-professional fields and some professional fields with low-precision requirements. Table 3 shows the convergence time and positioning accuracy of four data sets. The solutions of “smartphone single-frequency” were hard to converge to 1 m. The convergence time of “smartphone dual-frequency” was 102 min to 1 m, 107 min to 0.5 m, and 116 min to 0.2 m, respectively. Meanwhile, for the results of “receiver single-frequency”, the convergence time was 35 min to 1 m, and 158 min to 0.5 m. The results of “receiver dual-frequency” could reach 0.1 m accuracy in 301 min. Both kinds of receiver indicated a relatively higher positioning accuracy in dual-frequency.

And the RMS of position errors are calculated from [20]:

(39) $${\mathrm{RMS}}_{E}=\sqrt{\frac{1}{n}\sum_{i=1}^{n}\Delta E_{i}^{2}}$$

(40) $${\mathrm{RMS}}_{N}=\sqrt{\frac{1}{n}\sum_{i=1}^{n}\Delta N_{i}^{2}}$$

(41) $${\mathrm{RMS}}_{U}=\sqrt{\frac{1}{n}\sum_{i=1}^{n}\Delta U_{i}^{2}}$$

(42) $${\mathrm{RMS}}_{3D}=\sqrt{\frac{1}{n}\sum_{i=1}^{n}{\left(\Delta E_{i}^{2}+\Delta N_{i}^{2}+\Delta U_{i}^{2}\right)}^{2}}$$

where $RMS_{E}$, $RMS_{N}$, $RMS_{U}$ are RMS values of position errors in east, north and up directions, respectively, $RMS_{3D}$ is RMS values of 3D position errors, n is the number of positioning results, $\Delta E_{i}$, $\Delta N_{i}$ and $\Delta U_{i}$ are the position errors in the east, north and vertical components of the ith epoch.

The positioning accuracy of “smartphone single-frequency” could not be converged to 1 m. The RMS (Root Mean Square) positioning errors of “smartphone double-frequency” after convergence in the E, N, U directions were 21.8 cm, 4.1 cm, 11.0 cm. The positioning accuracy of “smartphone double-frequency” was at the same level of “receiver single-frequency”, and had an evident gap with “receiver dual-frequency” (Table 4).

The positioning accuracy of dual-frequency observations was better than that of single-frequency observations due to the distinct processing methods for ionospheric delay. The PPP of single-frequency observations implemented the ionospheric broadcast model, and the ionospheric errors were large. However, the process of dual-frequency observations applied the ionospheric-free combination, eliminating the first-order ionospheric delay. The positioning accuracy of receiver observations was higher than that of the smartphone observations because of the different antennas. The smartphone is equipped with a linearly polarized antenna, which has poor multipath suppression and irregular gain pattern. The receiver carries a geodetic-grade right-hand circularly polarized antenna, and the data sets can be acquired with very high quality.

**Figure 6:**
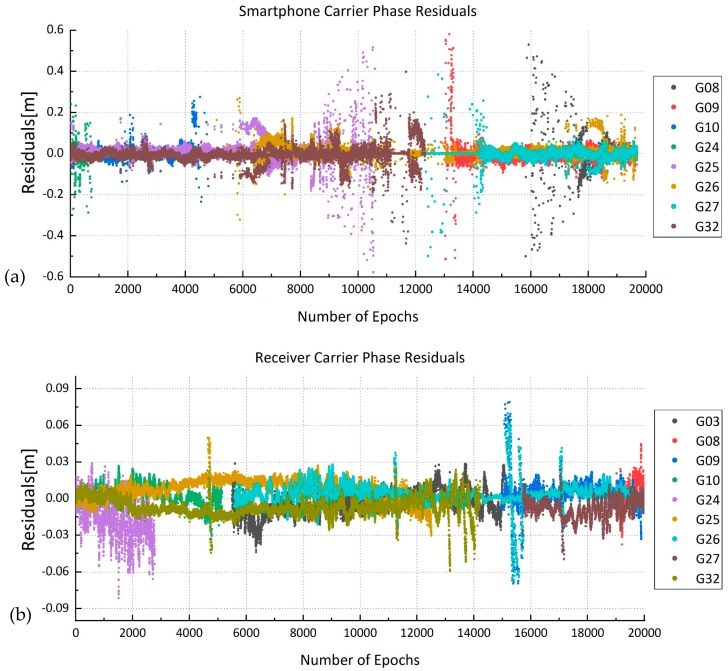
Carrier phase residuals of smartphone and receiver in static mode ((**a**) smartphone; (**b**) receiver).

The dual-frequency data sets had no results under several epochs due to the exclusion of some satellites with relatively larger carrier phase residuals (Figure 6 and Table 5). The carrier phase residuals of smartphone were higher than those of the receiver because larger measurement noises existed in the smartphone observations.

### 3.3. Kinematic Data Analysis

**Figure 7:**
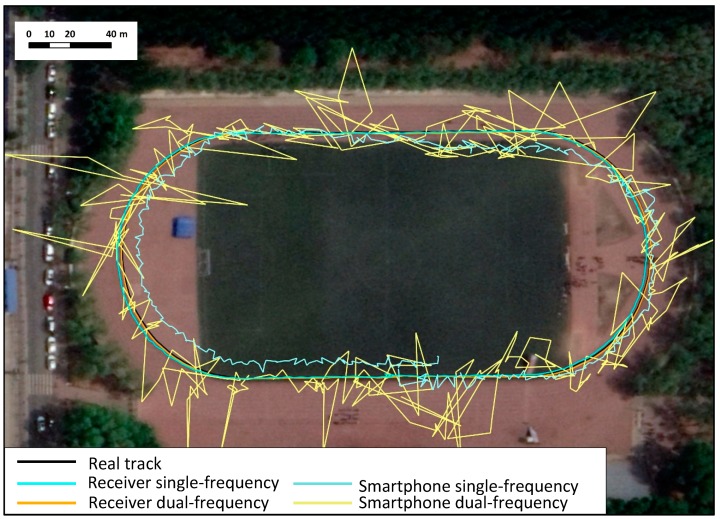
Kinematic positioning track in Google Earth.

The kinematic data was collected on the playground of Jilin University on October 16, 2018 (DOY 228 14:09-14:13UTC). The surrounding environment of the station is shown in Figure 7. The test was carried out along the runway track using a Mi 8 smartphone and a Hi-target geodetic GNSS receiver by means of a pedestrianly hand-held approach. The “real track” is the positioning result of geodetic receiver GPS L1/L2, Galileo E1/E5a observation data processed by RTKLIB PPP kinematic mode. In addition, the horizontal position errors $d_{i}$ are defined as the distances between “real track” and other tracks derived from four data sets using the following formula:

(43) $$d_{i}=\sqrt{\Delta E_{i}^{2}+\Delta N_{i}^{2}}$$

where $\Delta E_{i}$ and $\Delta N_{i}$ can be calculated from Equations (37) and (38).

**Figure 8:**
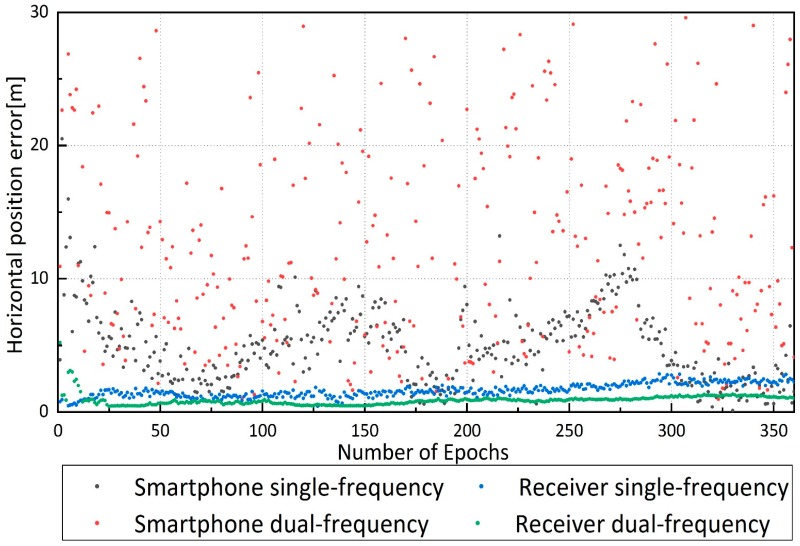
Horizontal position errors of four data sets in kinematic mode.

According to the data processing strategies in Table 1, the same four data sets in the previous section were processed in kinematic PPP mode. Figure 8 showed the horizontal position errors of four data sets in kinematic. The tracks of receiver data sets were slightly different from the real track, the “receiver dual-frequency” track was in good agreement with the real track, and the “receiver single-frequency” had an offset of approximately 1 m. The “smartphone dual-frequency” track had severe fluctuations within 4–5 m of the actual track with extreme offsets over 20 m, and the “smartphone single-frequency” track had an overall offset of 3–5 m.

**Figure 9:**
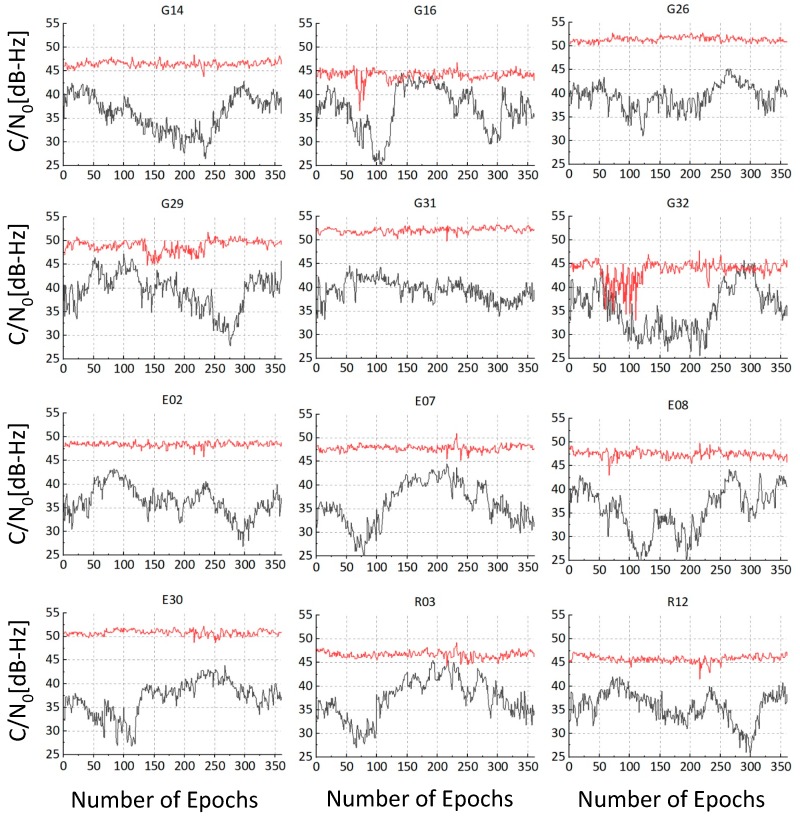
$C/N_{0}$ of GPS, Galileo and GLONASS satellites (Receiver: red lines; Smartphone: black lines).

**Figure 10:**
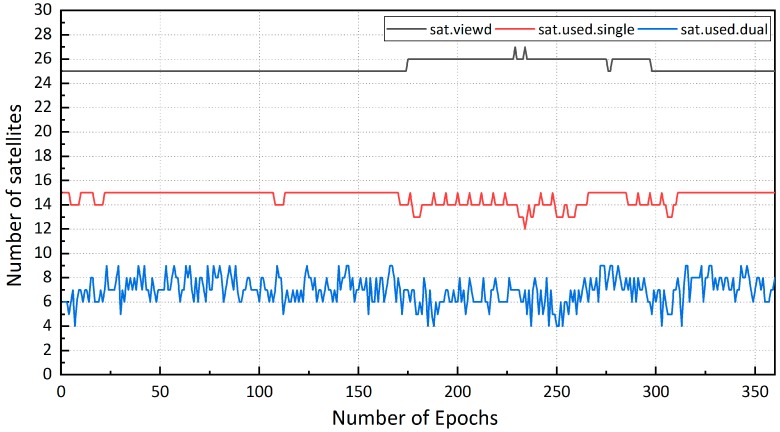
The number of satellites observed by smartphone.

In the kinematic mode, the positioning results of the receiver data sets were better than those of the smartphone data sets. 12 satellites of different satellite systems were selected in Figure 9, and the mean and STD of $C/N_{0}$ are summarized in Table 6. The smartphone $C/N_{0}$ (carrier-to-noise ratio) was approximately 10–15 dB-Hz lower than that of the receiver, indicating a poorer data quality due to the relatively larger observation noise of smartphone. The number of satellites observed by the smartphone was about 25, 15 of which were applied in single-frequency positioning, and 5 of which were applied in dual-frequency positioning. Although the antenna of the smartphone tracked many satellites, half of them could not be processed due to the low-quality data and low elevation angle (Figure 10). The track of “smartphone dual-frequency” was unstable compared to that of “smartphone single-frequency”. The observed number of satellites of the former was much lower than that of the latter. The large number of observed satellites of the “smartphone single-frequency” indicates relatively stable positioning results, and the lower number of observed satellites of “smartphone dual-frequency” indicates a small number of ionosphere-free observations, making it very difficult to get relatively stable and accurate positioning results.

## 4. Conclusions and Discussion

This paper analyzed the positioning performance of Mi 8 dual-frequency smartphone using a dual-frequency ionosphere-free combination PPP algorithm that considers the ISB and DCB. The results showed that the solutions of dual-frequency smartphone observations may achieve decimeter-level accuracy in static mode, which was comparable to the geodetic receiver in single-frequency mode, but a long convergence time was required for PPP. In the kinematic mode, the data quality of the dual-frequency smartphone was poor, and continuous positioning results were only obtained with difficulty, having an offset of 4–5 m from the actual track. Compared with the geodetic receiver, the positioning accuracy of double-frequency smartphone was 3–5 m in kinematic mode, which was worse than the single-frequency data of the smartphone. 

In recent years, many studies have been proposed to deal with different types of observations using various algorithms. The positioning results of pseudorange observations are generally worse than those of carrier phase observations. Positioning accuracy within 5 m can be achieved using the pseudorange double-difference algorithm to process carrier phase smoothing of the single-frequency code observations [14]. The positioning results obtained by single-frequency carrier phase observations usually vary with the different algorithms. For the relative positioning method with single-frequency phase observations in a fast static mode, the positioning accuracy is similar to the PPP used in this paper, but the solution of the former was highly affected by the baseline length and the base station type (virtual station or physical station) [15]. In addition to that, the combination of pseudorange, carrier phase and doppler observations might reach a positioning accuracy of 0.6 m and 1.4 m in the horizontal and vertical directions, respectively, using the time series differential algorithm with the SNR-dependent weighting method [17]. As for the PPP, the results of single-frequency phase observation showed an internal accuracy of 0.37 m and 0.51 m for the horizontal and vertical components, which was different from the external accuracy obtained in this paper [16].

PPP of a single smartphone can acquire high-precision positioning results without external equipment, and the procedure of data collection is low-cost and convenient. However, the convergence time of the smartphone was much longer than that of the geodetic receiver, which could be solved by the introduction of other algorithms in static positioning in the future. In the process of PPP used in this paper, the unfixed integer ambiguity led to an extended time for convergence, and solving ambiguity may be helpful in speeding up the convergence [31,32].

Smartphone hardware limitations, such as the crowded PCB (printed circuit board) space of the smartphone, the thermal noise generated during operation, and the linearly polarized antenna, are detrimental to the positioning results. The application of external antennas is one commonly used method to improve data quality and positioning accuracy [33].

In this paper, static and kinematic tests were carried out in low multipath environments such as with an open sky roof and in a playground. No experiment was conducted in a high multipath environment. The reason for this was that the smartphone cannot observe enough satellites due to its antenna, and it was difficult to obtain the positioning results after PPP processing.

The PPP accuracy of the dual-frequency smartphone can meet the requirements of most application scenarios, and can be used in semi-professional fields such as smart city, Internet of things (IoT), and smart transportation, as well as in professional fields with low precision requirements such as map updating and cadastral survey [34].

## Figures and Tables

**Table 1:** Data processing strategies.

Items	Strategies
Observations	Dual-frequency: GPS L1/L5, Galileo E1/E5a ionosphere-free combination of code and carrier phase Single-frequency: GPS L1, GLONASS L1, Galileo E1 code and carrier phase
Sampling rate	1 s
Elevation cutoff	10°
Observation weight	Elevation dependent weight
Orbits	WUM final orbits
Satellite clocks	WUM final clocks, 30 s interval
Tidal loadings	IERS conventions (2010) and FES2004 model [26]
Ionospheric delay	Dual-frequency: ionosphere-free combination mode Single-frequency: ionospheric broadcast model
Satellite antenna phase center correction	IGS14.atx
Receiver antenna phase center correction	IGS14.atx
Antenna phase wind-up correction	IGS model
DCB	CAS DCB file
Parameter estimation	Extended Kalman Filter (EKF)
Receiver coordinates	Estimate
Receiver clock	Estimate
Inter-system bias	Estimate
Ambiguities	Estimate, float
Tropospheric delay	Estimate ZTD and horizontal gradients

**Table 2:** STD of $MP_{1}$ and $MP_{5}$ in two cases.

Satellite	STD of MP_1_ under the Antenna	STD of MP_5_ under the Antenna	STD of MP_1_ on the Edge	STD of MP_5_ on the Edge
G24	0.68	0.70	0.78	0.71
G30	0.64	0.74	0.75	0.83
E02	0.71	0.72	N/A	N/A
E25	0.78	0.74	N/A	N/A
E04	N/A	N/A	0.67	0.76
E09	N/A	N/A	0.82	0.67

**Table 3:** The convergence time to different accuracies.

Data Sets	Positioning Accuracy (m)			
	1	0.5	0.2	0.1
smartphone single-frequency(min)	N/A	N/A	N/A	N/A
smartphone dual-frequency(min)	102	107	116	N/A
receiver single-frequency(min)	35	158	N/A	N/A
receiver dual-frequency(min)	66	107	272	301

**Table 4:** RMS positioning errors of four data sets.

Data Sets	East (cm)	North (cm)	Up (cm)
smartphone single-frequency	N/A	N/A	N/A
smartphone dual-frequency	21.8	4.1	11.0
receiver single-frequency	14.6	25.8	27.1
receiver dual-frequency	0.2	0.1	0.5

**Table 5:** Standard deviation of carrier phase residuals of smartphone and receiver (m).

Satellite	STD of Smartphone Carrier Phase Residual	STD of Receiver Carrier Phase Residual
G03	N/A	0.009
G08	0.073	0.010
G09	0.045	0.010
G10	0.032	0.008
G24	0.056	0.016
G25	0.051	0.008
G26	0.030	0.007
G27	0.032	0.007
G32	0.039	0.007

**Table 6:** Mean and standard deviation of $C/N_{0}$ of GPS, Galileo and GLONASS [dB-Hz].

Satellite	The Mean of Smartphone C/*N*_0_	The STD of Smartphone C/*N*_0_	The Mean of Receiver C/*N*_0_	The STD of Receiver C/*N*_0_
G14	36.07	3.77	46.53	0.59
G16	37.02	4.69	44.05	1.04
G26	39.29	2.62	51.41	0.52
G29	39.32	4.00	48.94	1.21
G31	39.46	2.14	52.06	0.56
G32	35.32	4.74	43.69	1.99
E02	36.41	3.17	48.39	0.49
E07	36.09	4.45	47.87	0.62
E08	35.63	4.80	47.46	0.75
E30	36.93	3.59	50.82	0.55
R03	37.38	4.24	46.67	0.68
R12	35.72	3.21	45.81	0.69
